# Autoimmunity Diseases of the Skin

**DOI:** 10.1155/2013/584597

**Published:** 2013-12-30

**Authors:** Jozélio Freire Carvalho, Paulo Ricardo Criado, Valéria Aoki, Yehuda Shoenfeld

**Affiliations:** ^1^Rheumatology Division, Aliança Medical Center, Rua das Violetas, 42, AP. 502, 41810-080 Salvador, BA, Brazil; ^2^Dermatology Division, Clinic Hospital from Sao Paulo University School of Medicine, 01239-040 Sao Paulo, SP, Brazil; ^3^Zabludowitcz Center for Autoimmune Diseases, Sheba Medical Center, Tel-Hashomer, Sackler Faculty of Medicine, Tel Aviv University, 52621 Tel-Hashomer, Israel

The tegumentary system becomes a scenario for immune responses. The knowledge of these conditions has led to induction of complementary animal models, better knowledge of the pathophysiology, and new tools [1] for diagnosis and therapy of these autoimmune skin disorders.

In this special issue from Autoimmune Diseases we have invited several papers to address the dermatological issue.

One paper of this issue analyzes epidemiological, clinical, and histopathological features of 56 patients with psoriasis in Africa. Severity, HIV associations, and diverse clinical aspects are discussed in this interesting paper. Another paper reviews the genetic aspect of psoriasis and also implications of the pharmacogenomics in predicting responses to therapeutical agents. An interesting recent review on psoriasis immunomodulation and treatment may be obtained at [2, 3]. One of the papers evaluated retrospectively in a large cohort of 155 patients with pemphigus vulgaris the incidence of infections. Interestingly, 94 cases of infection were detected and described. Pemphigus vulgaris was also studied in one paper with the aim to determine the involvement of the anal area in newly diagnosed pemphigus vulgaris patients.

Another paper of this issue reviewed p38 mitogen activated protein kinase (p38 MAPK) in the pathogenesis of pemphigus. P38 MAPK signaling plays a major role in the modulation of immune-mediated inflammatory responses and therefore has been linked with diverse autoimmune diseases.

One paper reviewed vascular alterations in patients with morphea, since it has been proposed that endothelial cell damage may represent the initial and pivotal step in the development of soft tissue changes in morphea.

Another paper of this collection has evaluated the role of interleukin-8 in patients with dermatitis herpetiformis associated with gluten-sensitive enteropathy. It brings a new methodology for treating arthritis. The rational consists that small bowel as a mucosal immune system, responding to gluten ingestion with high levels of interleukin-8, and that the mucosal immune response was associated with the development of the skin lesions in dermatitis herpetiformis. It was previously demonstrated in the Caucasian and it was for the first time shown in the present study in Japanese patients.
